# Antibody-drug conjugates targeting CD248 inhibits liver fibrosis through specific killing on myofibroblasts

**DOI:** 10.1186/s10020-022-00460-1

**Published:** 2022-03-22

**Authors:** Shaojie Liu, Donghui Han, Chao Xu, Fa Yang, Yu Li, Keying Zhang, Xiaolong Zhao, Jiayu Zhang, Tong Lu, Shiqi Lu, Changhong Shi, Rui Zhang, An-Gang Yang, Aizhi Zhao, Weijun Qin, Bo Yang, Weihong Wen

**Affiliations:** 1 Department of Urology, Xijing Hospital, Fourth Military Medical University, Xi’an, 710032 China; 2 Institute of Medical Research, Northwestern Polytechnical University, Xi’an, 710072 China; 3 Laboratory Animal Center, Fourth Military Medical University, Xi’an, 710032 China; 4 State Key Laboratory of Cancer Biology, Department of Immunology, Fourth Military Medical University, Xi’an, 710032 China; 5 OriMAbs Ltd., 250 Corporate Blvd, Suite C, Newark, DE 19702 USA

**Keywords:** Liver fibrosis, Myofibroblasts, CD248, Antibody-drug conjugate, IgG78-DM1

## Abstract

**Background:**

Chronic liver injury induces pathological repair, resulting in fibrosis, during which hepatic stellate cells (HSCs) are activated and transform into myofibroblasts. CD248 is mainly expressed on myofibroblasts and was considered as a promising target to treat fibrosis. The primary aim of this study was to generate a CD248 specific antibody-drug conjugate (ADC) and evaluate its therapeutic efficacy for liver fibrosis and its safety in vivo.

**Methods:**

CD248 expression was examined in patients with liver cirrhosis and in mice with CCl_4_-induced liver fibrosis. The ADC IgG78-DM1, which targets CD248, was prepared and its bioactivity on activated primary HSCs was studied. The anti-fibrotic effects of IgG78-DM1 on liver fibrosis were evaluated in CCl_4_-induced mice. The reproductive safety and biosafety of IgG78-DM1 were also evaluated in vivo.

**Results:**

CD248 expression was upregulated in patients with liver cirrhosis and in CCl_4_-induced mice, and was mainly expressed on alpha smooth muscle actin (α-SMA)^+^ myofibroblasts. IgG78-DM1 was successfully generated, which could effectively bind with and kill CD248^+^ activated HSCs in vitro and inhibit liver fibrosis in vivo. In addition, IgG78-DM1 was demonstrated to have qualified biosafety and reproductive safety in vivo.

**Conclusions:**

Our study demonstrated that CD248 could be an ideal target for myofibroblasts in liver fibrosis, and CD248-targeting IgG78-DM1 had excellent anti-fibrotic effects in mice with liver fibrosis. Our study provided a novel strategy to treat liver fibrosis and expanded the application of ADCs beyond tumors.

**Graphic Abstract:**

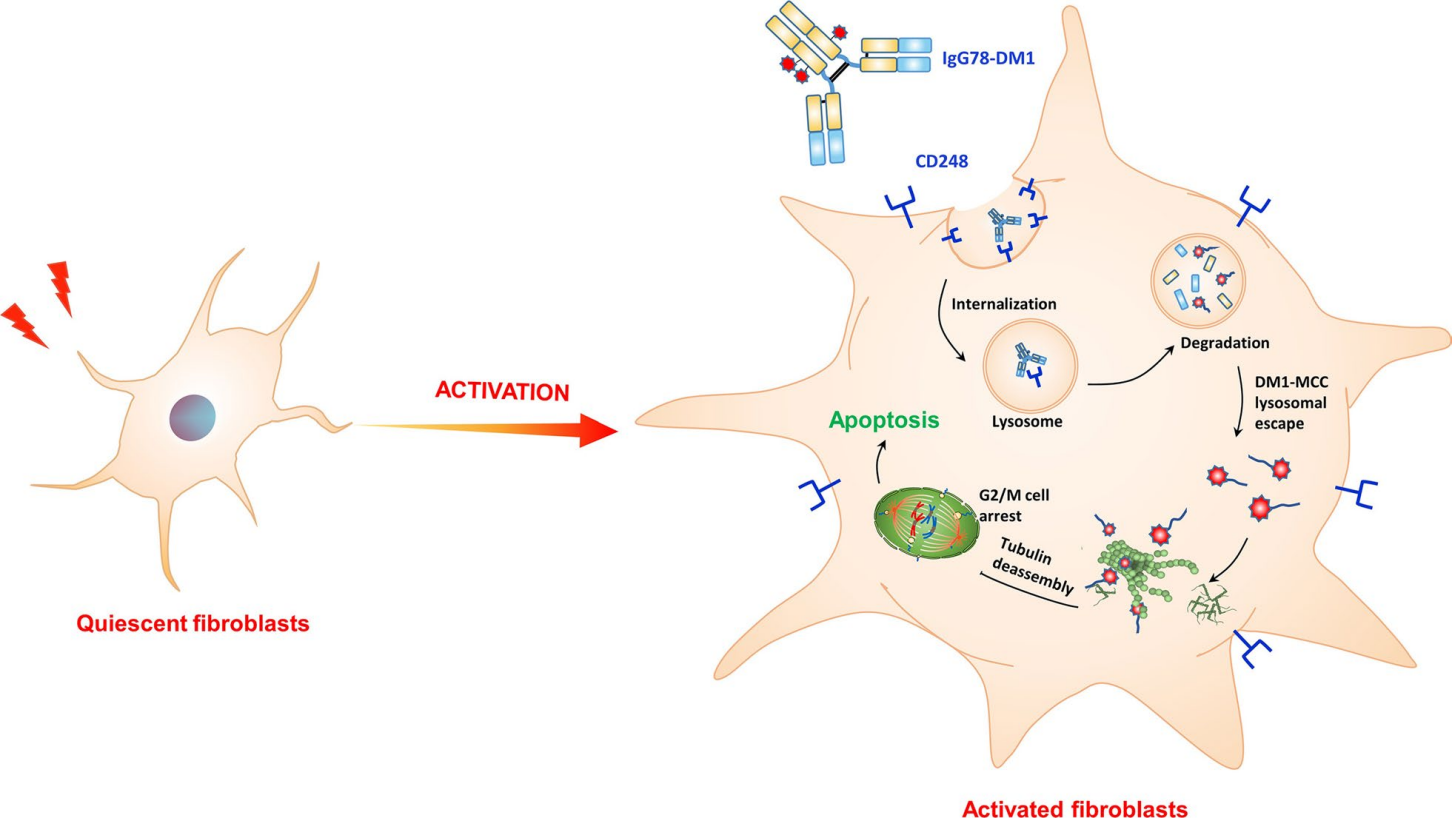

**Supplementary Information:**

The online version contains supplementary material available at 10.1186/s10020-022-00460-1.

## Introduction

Fibrosis is the pathological repair response of a tissue to chronic injury, which is characterized by excessive accumulation and deposition of extracellular matrix (ECM) proteins, leading to organ dysfunction. Fibrosis can be triggered by many factors, including viral and parasitic infections, autoimmune inflammation, and drugs/toxic effects, and different organs can be affected, resulting in diseases such as liver fibrosis (Bataller and Brenner 2005; Distler et al. 2019). Liver fibrosis can lead to liver cirrhosis, decompensation of liver function, and ultimately, death. However, there is no effective treatment strategy for liver fibrosis, and existing treatment strategies cannot meet clinical needs (Friedman et al. 2013; Schuppan and Kim 2013). Thus, there is an urgent need to explore novel anti-fibrotic treatment strategies.

The occurrence of liver fibrosis is accompanied by chronic inflammation, during which the levels of pro-inflammatory cytokines are upregulated and released to activate hepatic stellate cells (HSCs), among which transforming growth factor-β (TGF-β) and platelet-derived growth factor (PDGF) are the most potent pro-fibrotic cytokines (Bonner 2004; Meng et al. 2016; Stewart et al. 2018). Activation of HSCs is considered as the pivotal event driving and aggravating liver fibrosis. During chronic liver injury, the resident quiescent HSCs are activated and transform into highly proliferative, motile, and contractile myofibroblasts, which are the main source of ECM proteins, occupying up to 90% of the fibrotic liver (Bataller and Brenner 2005; Higashi et al. 2017). Activated HSCs have been considered as a target for anti-fibrotic therapy because of their central role in liver fibrosis.

The resistance of myofibroblasts to apoptosis is considered critical for the aberrant accumulation of these cells in fibrotic diseases, and different strategies to induce apoptosis of myofibroblasts have been examined in different fibrotic diseases (Hinz and Lagares 2020). Recently, Aghajanian et al. reported that in an angiotensin II and phenylephrine (AngII/PE)-induced cardiac fibrosis model, fibroblast activation protein (FAP) was expressed specifically in activated cardiac fibroblasts. In addition, they confirmed that the specific killing of activated cardiac fibroblasts by FAP-specific chimeric antigen receptor (CAR) T cells could reduce cardiac fibrosis and restore cardiac function in mice (Aghajanian et al. 2019). That report indicated that specific killing of myofibroblasts could be an effective strategy to inhibit tissue fibrosis. However, developing strategies to kill myofibroblasts specifically in liver fibrosis is limited by the lack of effective therapeutic targets (Bansal et al. 2016).

CD248, also known as endosialin or tumor endothelial marker 1 (TEM1), is a type I transmembrane glycoprotein (Christian et al. 2001). Previously, CD248 was found to be expressed specifically in pericytes, cancer-associated fibroblasts (CAFs), and some tumor cells, such as sarcomas, with very low or limited expression in normal tissue; therefore, CD248 has been considered as a specific target for cancer therapy (Bagley et al. 2008; MacFadyen et al. 2005; O'Shannessy et al. 2016; Rouleau et al. 2008). Later studies showed that CD248 expression was also upregulated specifically in liver fibrosis, and CD248 was expressed mainly in activated HSCs, but not in quiescent HSCs, indicating that CD248 could be an effective therapeutic target for liver fibrosis (Wilhelm et al. 2016). To realize specific killing of activated HSCs, we aimed to generate an antibody-drug conjugate (ADC) by conjugating a CD248-specific antibody, IgG78, with the microtubule inhibitor mertansine (DM1) via non-cleavable succinimidyl 4-(*N*-maleimidomethyl) cyclohexane-1-carboxylate (SMCC) linker.

In this study, we first examined CD248 expression in the liver tissue of patients with hepatic cirrhosis and in carbon tetrachloride (CCl_4_)-induced liver fibrosis in C57BL/6 mice, and confirmed that CD248 was mainly expressed in alpha smooth actin (α-SMA)^+^ myofibroblasts. We then demonstrated that CD248 expression was increased in TGF-β activated primary HSCs and freshly isolated HSCs from CCl_4_-induced mice. The ADC IgG78-DM1 was then generated through conjugating IgG78 with DM1 via the non-cleavable SMCC linker. After confirming its binding affinity and specific cytotoxicity toward CD248 positive HSCs in vitro, we verified that IgG78-DM1 could alleviate CCl_4_-induced liver fibrosis in vivo. The biosafety and reproductive safety of IgG78-DM1 were also examined in vivo. The present study demonstrated that CD248 is an ideal target for anti-fibrotic therapy and specific killing of CD248^+^ myofibroblasts using IgG78-DM1 could be a novel and effective strategy to treat liver fibrosis.

## Materials and methods

### Human tissue samples and animal models

A human liver tissue microarray (HC-Liv00006) was purchased from Avila Biotech (Xi’an, China). Male C57BL/6 mice were purchased from experimental animal center, Fourth Military Medical University (Xi’an, China). Liver fibrosis in the mice was induced by intraperitoneal injection of CCl_4_ (2.5 mL/kg body weight, dissolved in olive oil at the ratio of 1:9) twice a week for 6 weeks (n = 5 mice in each group). Mice were sacrificed 3 days after the final injection.

### Histological staining

Mouse liver tissue slides were prepared as 5 μm-thick paraffin sections and 8 μm-thick frozen sections. Hematoxylin and eosin (H&E) staining and Masson staining were performed by Servicebio Co., Ltd. (Wuhan, China) The primary antibodies used for immunohistochemistry (IHC) staining and immunofluorescent (IF) staining were as follows: anti-human CD248 (#ab67273, Abcam, Cambridge, UK), anti-mouse CD248 (#18160-1-AP, Proteintech, Rosemont, IL, USA), anti-mouse α-SMA (#ab124964, Abcam), Lysotracker™ Red DND 99 (#L7528, Invitrogen, Waltham, MA, USA), Alexa Fluor 488 conjugated α-tubulin (11H10) Rabbit monoclonal antibody (mAb) (#5063, Cell Signaling Technology, Danvers, MA, USA). Apoptotic cells were stained using a Fluorescein isothiocyanate (FITC) terminal deoxynucleotidyl transferase nick-end-labeling (TUNEL) Cell Apoptosis Detection Kit (#G1501-50, Servicebio). At 24 h after a single injection of IgG78-DM1, liver tissues were isolated and used to examine the co-location of IgG78-DM1 and CD248 by dual IF staining and cell apoptosis by TUNEL assay. Images were captured under a fluorescence microscope or a confocal laser scanning microscope. Quantification was performed according to the percentage and intensity in IHC staining and the percentage of the positive area in the IF staining using Image J v1.52a (NIH, Bethesda, MD, USA).

### Purification of IgG78 and preparation of IgG78-DM1

A plasmid containing the DNA sequence encoding IgG78 was transiently transfected into HEK293F cells using the FreeStyle™ MAX Reagent (#16447100, Gibco, Grand Island, NY, USA) and cultured for 7 days before supernatants were collected. IgG78 was purified from the supernatant using a HiTrap Protein A FF column (#28-9343-88, GE Healthcare, Chicago, IL, USA) and an AKTA fast protein liquid chromatography (FPLC) Protein Purifier (#03009481, GE Healthcare). After being dialyzed in conjugating buffer (50 mM Trisodium phosphate, 50 mM NaCl, 2 mM EDTA dissolved in 1 L ddH_2_O, PH = 7.2), IgG78 was then conjugated with SMCC-DM1 dissolved in *N*, *N*-dimethylacetamide (DMA) at room temperature for 3 h. Then, IgG78-DM1 was dialyzed for 24 h to replace the solvent with phosphate-buffered saline (PBS). The ratio of DM1 to IgG78 was calculated as follows (C_DM1_ = Molar concentration of DM1; ε_ab_ = Molar extinction coefficient of IgG78, ε_ab@252_ = 82,880 M^−1^ cm^−1^, ε_ab@280_ = 224,000 M^−1^ cm^−1^; ε_DM1_ = Molar extinction coefficient of DM1, ε_DM1@252_ = 26,790 M^−1^ cm^−1^, ε_DM1@280_ = 5700 M^−1^ cm^−1^):

$${C}_{DM1}=\frac{A252-A280*\frac{{\varepsilon }_{ab@252}}{{\varepsilon }_{ab@280}}}{{\varepsilon }_{DM1@252}-{\varepsilon }_{DM1@280}*\frac{{\varepsilon }_{ab@252}}{{\varepsilon }_{ab@280}}}$$

$$\frac{DM1}{IgG78} ratio=\frac{{C}_{DM1}}{{C}_{IgG78}}$$

The heavy and light chains of IgG78-DM1 was resolved by 10% sodium dodecyl sulfate polyacrylamide gel electrophoresis (SDS-PAGE) for Coomassie blue staining and western blotting.

### Culture of cell lines and isolation of primary HSCs

The mouse macrophage cell line RAW264.7 and mouse hepatocyte cell line AML12 were purchased from American type culture collection (ATCC; Manassas, VA, USA). The mouse HSC cell line JS-1 was purchased from Fenghbio Co., Ltd (Changsha, China). Cells were maintained in Dulbecco’s modified Eagle’s medium (DMEM) medium supplemented with 10% fetal bovine serum (FBS) (#A3161002C, Gibco) and 1% penicillin–streptomycin (#P1400, Solarbio). Primary HSCs were isolated from C57BL/6 mice according to a previously described protocol (Castello-Cros and Cukierman 2009; Mederacke et al. 2015). Primary HSCs were maintained in fibroblast-specific medium (#2301, Sciencell, Carlsbad, CA, USA), and were activated by TGF-β1 (#AF-100-21C, Peprotech, Rocky Hill, NJ, USA) for 48 h before further analysis.

### Flow cytometry

Primary HSCs were incubated with Alexa fluor 647-conjugated Vimentin (D21H3) rabbit mAb (#9856, Cell Signaling Technology), anti-mouse CD248 primary antibody (#ab217535, Abcam) or 100 nM IgG78-DM1 at 4 °C for 30 min, followed by incubation with FITC anti-rabbit IgG Fc (Abcam, #ab6717) and FITC anti-human IgG Fc (Abcam, #96907) at 4 °C for 30 min in the dark before flow cytometry analysis. Hepatocytes and macrophages were incubated with 100 nM IgG78-DM1 at 4 °C for 30 min, followed by incubation with Phycoerythrin (PE) anti-human IgG Fc (BioLegend, San Diego, CA, USA #410708).

### Cellular ELISA and CCK-8 assay

Activated HSCs were counted to adjust the cell density to 1 × 10^4^/mL, and the cells were plated at 200 μL/well in 96-well plates coated with 2% Gelatin. Then, HSCs were washed and incubated with IgG78-DM1 or control hIgG-DM1 (DM1 conjugated to non-specific human IgG) at 4 °C for an enzyme-linked immunosorbent assay (ELISA) or at 37 °C for a CCK-8 (Cell Counting Kit-8) assay. For ELISA, HSCs were then incubated with peroxidase-labeled secondary antibody (#A21050, Abbkine, Wuhan, China) for 1 h at 4 °C before colorimetric signals were developed by incubation with 3,3ʹ,5,5ʹ-tetramethylbenzidine (TMB; #P0209, Beyotime, Jiangsu, China) and stopped by 2 M H_2_SO_4_ for 15 min (#P0215, Beyotime). The CCK-8 kit (#BS350A, Biosharp, Anhui, China) was used to count live HSCs. Absorbance was then measured at 450 nm using a microplate reader, and the binding affinity and IC50 of IgG78-DM1 were calculated with GraphPad Prism 8 (GraphPad Inc., La Jolla, CA, USA).

### Quantitative real-time reverse transcription polymerase chain reaction (qRT-PCR)

Total RNA was extracted using the Trizol reagent, and reverse transcription (RT) was performed using PrimeScript™ RT Master Mix (#RR036A, Takara, Shiga, Japan). The resultant cDNA was quantified using qPCR, performed using a TB Green^®^ Premix Ex Taq™ II kit (#RR820A, Takara). The primers used for qPCR are listed in Table 1.

**Table 1:** Primers used for qRT-PCR analysis

Target	Forward primer	Reverse primer
*Cd248*	CTCAACCAACTATCCCCAAGTC	GCCTGGGTTCTGATACCTGG
*Acta2*	CCGCCATGTATGTGGCTATT	CAGTTGTACGTCCAGAGGCATA
*Col1a1*	AGACATGTTCAGCTTTGTGGAC	GCAGCTGACTTCAGGGATG
*Tgfbr1*	CACAGAGTGGGAACAAAAAGGT	CCAATGGAACATCGTCGAGCA
*Pdgfrα*	ATGAGAGTGAGATCGAAGGCA	CGGCAAGGTATGATGGCAGAG

### Western blotting

Cells and tissues were lysed using Radioimmunoprecipitation assay (RIPA) buffer (#P0013B, Beyotime) and the protein was quantified using a bicinchoninic acid (BCA) protein assay kit (#P0010, Beyotime). Proteins were subjected to 8% SDS-PAGE and then transferred to a PVDF membrane for western blotting. The primary antibodies used were: anti-mouse CD248 (#ab48185, Abcam), anti-mouse α-SMA (#ab124964, Abcam), anti-mouse collagen I (#ab34710, Abcam), anti-mouse glyceraldehyde-3-phosphate dehydrogenase (GAPDH; #10494-1-AP, Proteintech).

### Statistical analysis

All data are presented as the mean ± standard deviation (SD). Before analysis, quantitative data were tested for normality and homogeneity of variance using GraphPad Prism 8. Statistical analyses were performed using Student’s test or linear correlation. Differences were considered significant when p < 0.05.

## Results

### CD248 was mainly expressed on myofibroblasts in liver fibrosis

**Fig. 1:**
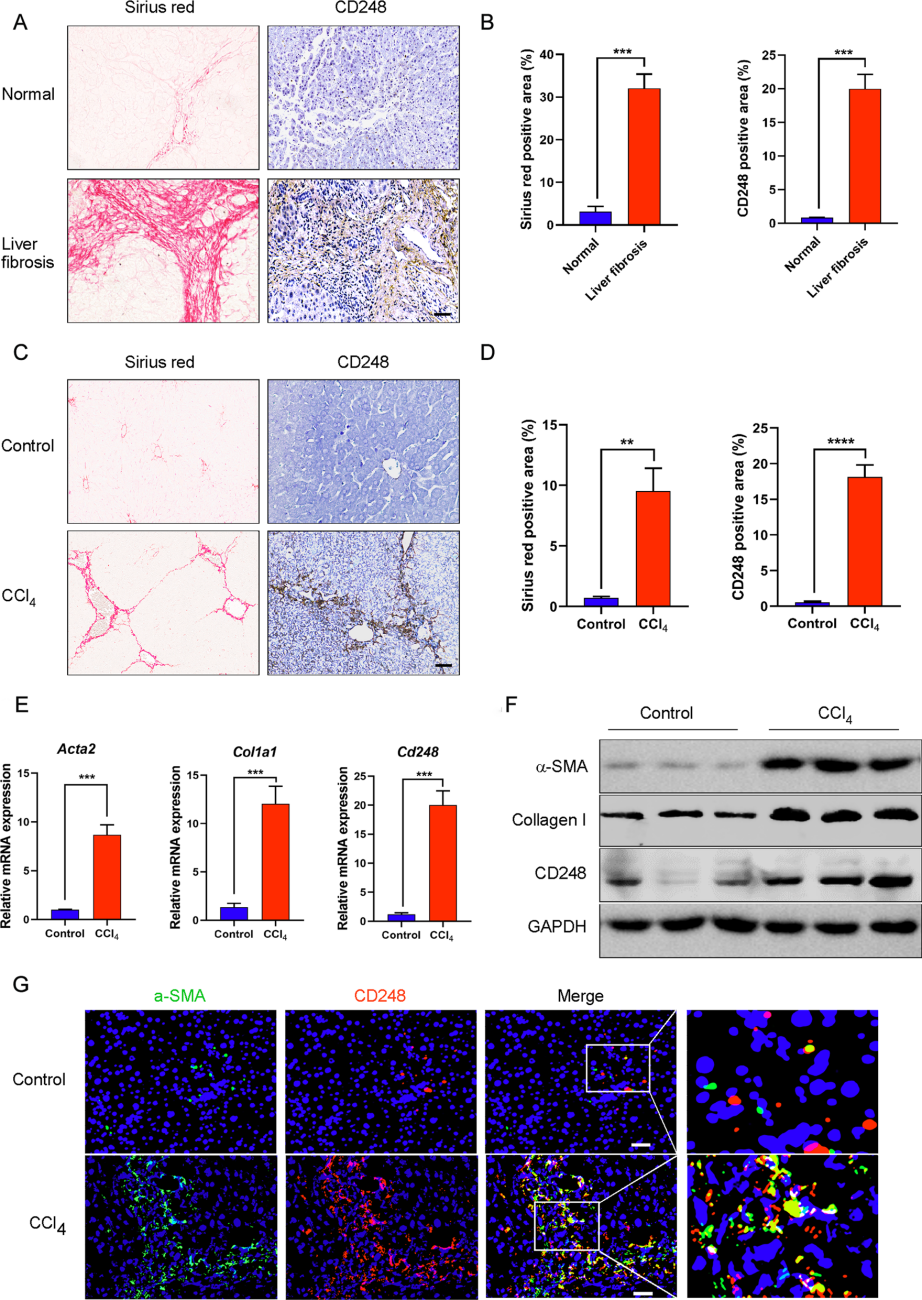
CD248 was mainly expressed on myofibroblasts in liver fibrosis. **A** Sirius red staining and IHC staining of CD248 in the liver tissue of 9 heathy controls and 40 patients with liver cirrhosis. **B** Quantitative analysis of the data in **A**. **C** Sirius red staining and IHC staining of CD248 in the liver tissue of CCl_4_-induced mice. **D** Quantitative analysis of the data in **C**. **E**, **F** qRT-PCR and western blotting analysis to show the increased expression of α-SMA, Collagen I, and CD248 in the liver tissues of CCl_4_-induced mice (n = 3 in **B**–**F**). **G** IF staining images showing the colocalization of CD248 and α-SMA in the liver tissue of CCl_4_-induced mice. Representative images are shown. Scale bar, 100 μm; **p < 0.01, ***p < 0.001. *IHC* immunohistochemistry, *qRT-PCR* quantitative real-time reverse transcription polymerase chain reaction, *α-SMA* alpha smooth muscle actin, *IF* immunofluorescence

To determine CD248 expression in liver fibrosis, we performed Sirius red and IHC staining for CD248 in the tissue array, which contained 40 cirrhotic liver tissues and 9 healthy controls. The results showed CD248 expression was significantly increased in human cirrhotic livers (Fig. 1A, B, p < 0.001) and the CD248 staining level correlated positively with the severity of liver fibrosis (Additional file 1: Fig. S1A, B, *p* < 0.0001). Next, we examined CD248 expression in 6-week CCl_4_-induced mice. IHC staining showed that CD248 levels were markedly upregulated in liver fibrosis (Fig. 1C, D, p < 0.0001). We then confirmed the upregulated expression of *Acta2* (encoding actin alpha 2, smooth muscle) and *Col1a1* (encoding collagen type I alpha 1 chain), which are fibrosis-related genes, and *Cd248* using qRT-PCR; and assessed α-SMA, Collagen I, and CD248 protein levels using western blotting (Fig. 1E, F). IF staining was also performed to localize CD248 expression. As shown in Fig. 1G, CD248 was mainly expressed on α-SMA^+^ myofibroblasts in liver fibrosis.

### CD248 expression was upregulated on activated primary HSCs

**Fig. 2:**
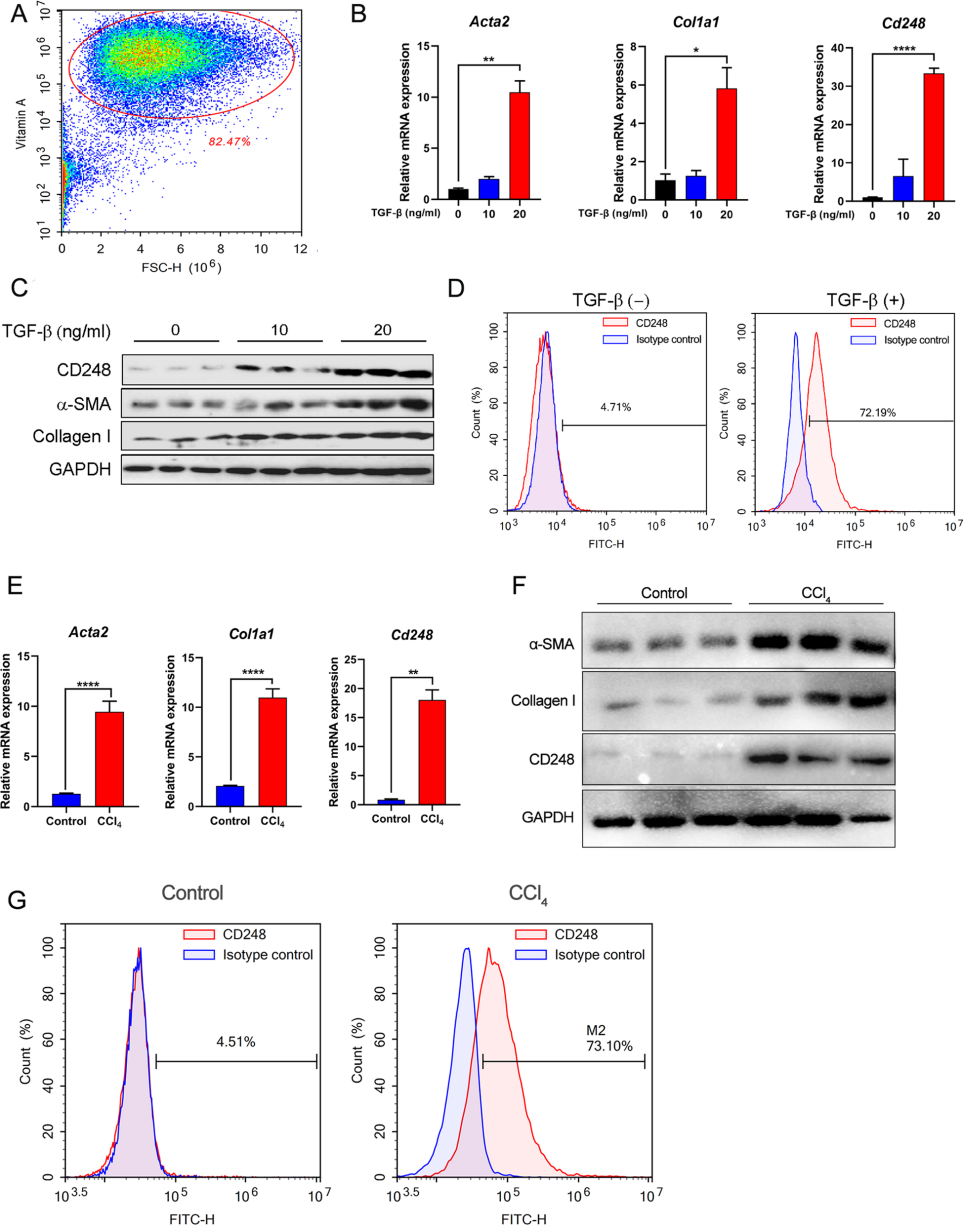
CD248 expression was upregulated on activated primary HSCs. **A** Flow cytometry to show the auto-fluorescent Vitamin A, which is a marker of HSCs. **B**, **C** qRT-PCR and western blotting analysis to show the increased expression of α-SMA, Collagen I and CD248 in TGF-β1-activated primary HSCs. **D** Flow cytometry showing the increased expression of CD248 in TGF-β1-activated primary HSCs (n = 3 in **A**–**D**). **E**, **F** qRT-PCR and western blotting analysis showing the increased expression of α-SMA, Collagen I, and CD248 in freshly isolated primary HSCs from CCl_4_-induced mice. **G** Flow cytometry showing the increased expression of CD248 in freshly isolated primary HSCs from control and CCl_4_-induced mice (n = 3 in **E**–**G**). Representative images are shown. *p < 0.05, **p < 0.01, ****p < 0.0001. *HSC* hepatic stellate cell, *qRT-PCR* quantitative real-time reverse transcription polymerase chain reaction, *α-SMA* alpha smooth muscle actin, *TGF-β* transforming growth factor beta

In CCl_4_-induced liver fibrosis, HSCs are the main source of myofibroblasts. To confirm the expression of CD248 in activated HSCs, we isolated primary HSCs from healthy mouse livers. Freshly isolated HSCs were subjected to flow cytometry analysis to observe the auto-fluorescence of Vitamin A, which is a marker of HSCs (Fig. 2A). HSCs are normally considered to be in a quiescent state, and their CD248 expression was low. However, after being activated by the potent pro-fibrotic cytokine TGF-β, the expression levels of CD248 and fibrosis-related genes, such as those encoding α-SMA and Collagen I, were upregulated significantly in the mouse HSC cell line JS-1 (Additional file 2: Fig. S2A–C). Similar results were also observed in freshly isolated HSCs (Fig. 2B, C). The increased CD248 expression was confirmed using flow cytometry (Fig. 2D). In addition, we isolated primary HSCs from CCl_4_-induced fibrotic mice and control mice to examine the expression of CD248 and other fibrosis-related genes. As shown in Fig. 2E–G, compared with those in the control mice, the expression levels of CD248 and other fibrosis-related genes were significantly increased in CCl_4_-induced fibrotic mice. These results confirmed that CD248 expression was upregulated in activated primary HSCs both in vitro and in vivo.

### Purification of IgG78 and preparation of IgG78-DM1

**Fig. 3:**
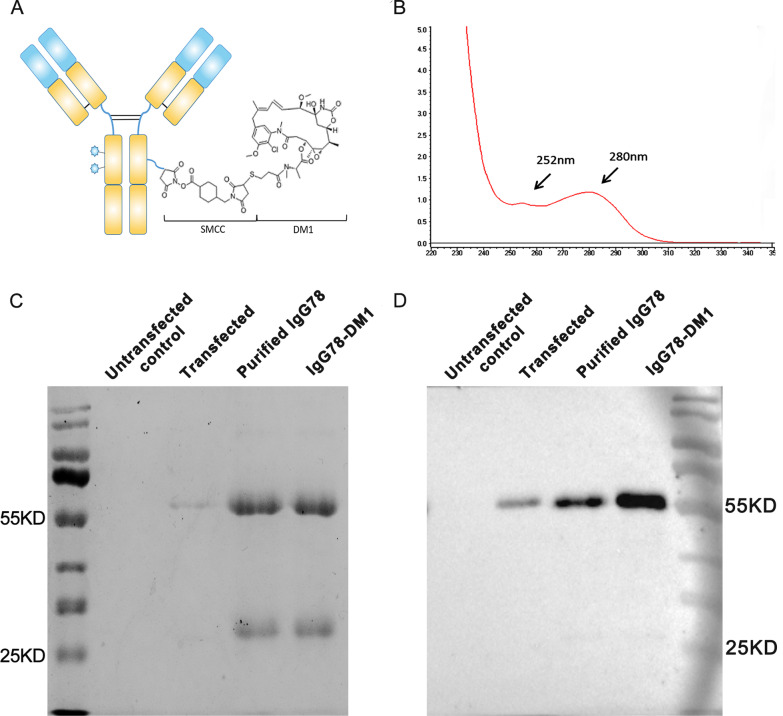
Purification of IgG78 and preparation of IgG78-DM1. **A** Schematic diagram of IgG78-DM1. **B** Characterization of IgG78-DM1 using a Nanodrop spectrophotometer to show the absorption peak of DM1 and IgG78 at 252 nm and 280 nm, respectively. **C** SDS-PAGE showing the expression of purified IgG78 and IgG78-DM1. **D** Western blotting to showing the purified IgG78 and generated IgG78-DM1. *DM1* Mertansine, *SDS-PAGE* sodium dodecyl sulfate polyacrylamide gel electrophoresis

To explore whether specific killing of myofibroblasts could effectively alleviate liver fibrosis, we first expressed and purified the fully human antibody IgG78, which specifically recognizes CD248. Then, we generated the ADC, IgG78-DM1, in which IgG78 was linked to DM1 via the stable thioether bond linker SMCC (Fig. 3A). The concentration of IgG78-DM1 was measured using a Nanodrop spectrophotometer, and the drug antibody ratio (DAR) of DM1 to IgG78 was calculated as 3.09, indicating that each IgG78 was conjugated with three DM1 molecules (Fig. 3B). To examine whether the conjugation process might destroy the structure and stability of IgG78, we performed SDS-PAGE, which showed that both IgG78 and IgG78-DM1 could be dissociated into heavy and light chains without other bands, indicating that the structure and stability of IgG78 was not influenced by the conjugation process (Fig. 3C). The stability of IgG78 was also confirmed by western blotting after DM1 conjugation, which showed that both IgG78 and IgG78-DM1 had a clear heavy chain, indicating that the DM1 conjugation did not influence the stability of IgG78 (Fig. 3D).

### Bioactivity of IgG78-DM1 toward primary HSCs in vitro

**Fig. 4:**
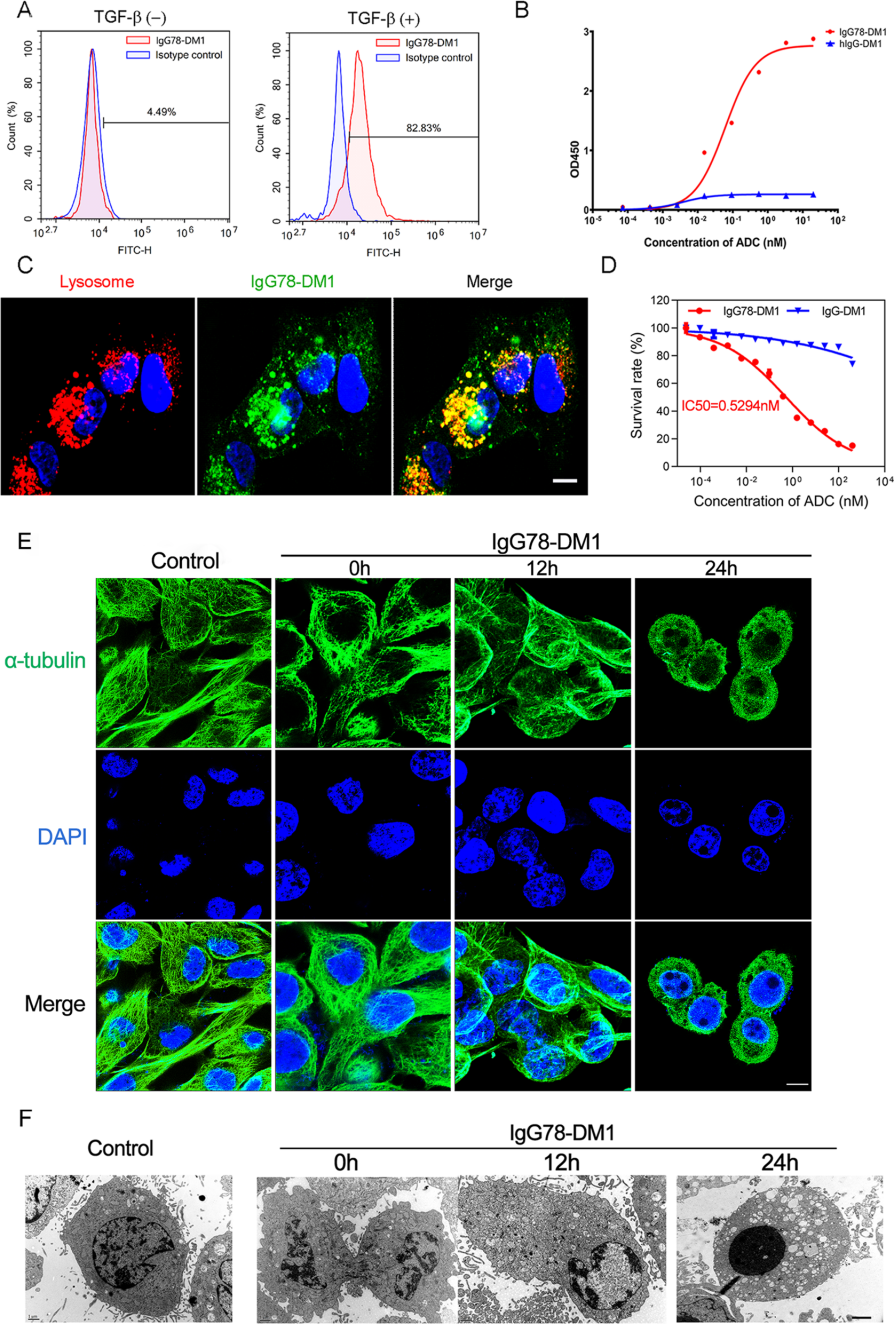
Characterization of IgG78-DM1 and its bioactivity toward primary HSCs in vitro. **A** Flow cytometry showing the binding of IgG78-DM1 with TGF-β1-activated primary HSCs. **B** ELISA to show the binding affinity of IgG78-DM1 with TGF-β1 activated primary HSCs. **C** IF staining images showing the co-localization of IgG78-DM1 and lysosomes. Scale bar, 50 μm. **D** CCK-8 assay showing the effective killing of activated primary HSCs by IgG78-DM1. **E** Laser confocal microscopy images showing that IgG78-DM1 could destroy the assembly of α-tubulin in activated primary HSCs. Scale bar, 2 μm. **F** Transmission electron microscopy images showing that IgG78-DM1 could induce obvious apoptosis in activated primary HSCs. Three independent experiments were performed and analysed. Representative images are shown. *DM1* Mertansine, *HSC* hepatic stellate cell, *TGF-β* transforming growth factor beta, *ELISA* enzyme-linked immunosorbent assay, *IF* immunofluorescence, *CCK-8* cell counting kit-8

To study the bioactivity of IgG78-DM1, we examined its binding with primary HSCs using flow cytometry. The results showed that IgG78-DM1 could bind effectively to activated HSCs, which have upregulated CD248 expression (Fig. 4A). Then, we examined the binding affinity of IgG78-DM1 using a cellular ELISA, which showed that IgG78-DM1 had relatively high binding affinity with activated HSCs, with a Kd value of 0.061 nM (Fig. 4B). Dual IF staining was then carried out to confirm whether IgG78-DM1 could internalize into activated HSCs, which showed that IgG78-DM1 co-localized mainly with lysosomes, indicating that it could be internalized into CD248^+^ HSCs (Fig. 4C).

The CCK-8 assay was then used to assess the specific killing of activated HSCs by IgG78-DM1, which showed that IgG78-DM1 had obvious cytotoxicity toward activated HSCs, with an IC50 of 0.5294 nM (Fig. 4D). Flow cytometry and CCK-8 assay were also used to examine the binding and cytotoxicity of IgG78-DM1 toward mouse hepatocytes or macrophages. The results showed that IgG78-DM1 could not bind with hepatocytes or macrophages and had no obvious cytotoxicity toward them (Additional file 3: Fig. S3A–D). These results further demonstrated the specificity of IgG78-DM1. We also evaluated the intracellular microtubule network in activated HSCs. As shown in Fig. 4E, the cells started to shrink and the microtubule structure was gradually destroyed after IgG78-DM1 treatment. Transmission electron microscopy observation revealed the condensation and fragmentation of nuclear chromatin in activated HSCs after IgG78-DM1 treatment (Fig. 4F). These results demonstrated that IgG78-DM1 has a high binding affinity for CD248^+^ HSCs and could effectively induce their apoptosis in vitro.

### IgG78-DM1 could alleviate liver fibrosis in CCl_4_-induced fibrotic mice through specific killing of myofibroblasts

**Fig. 5:**
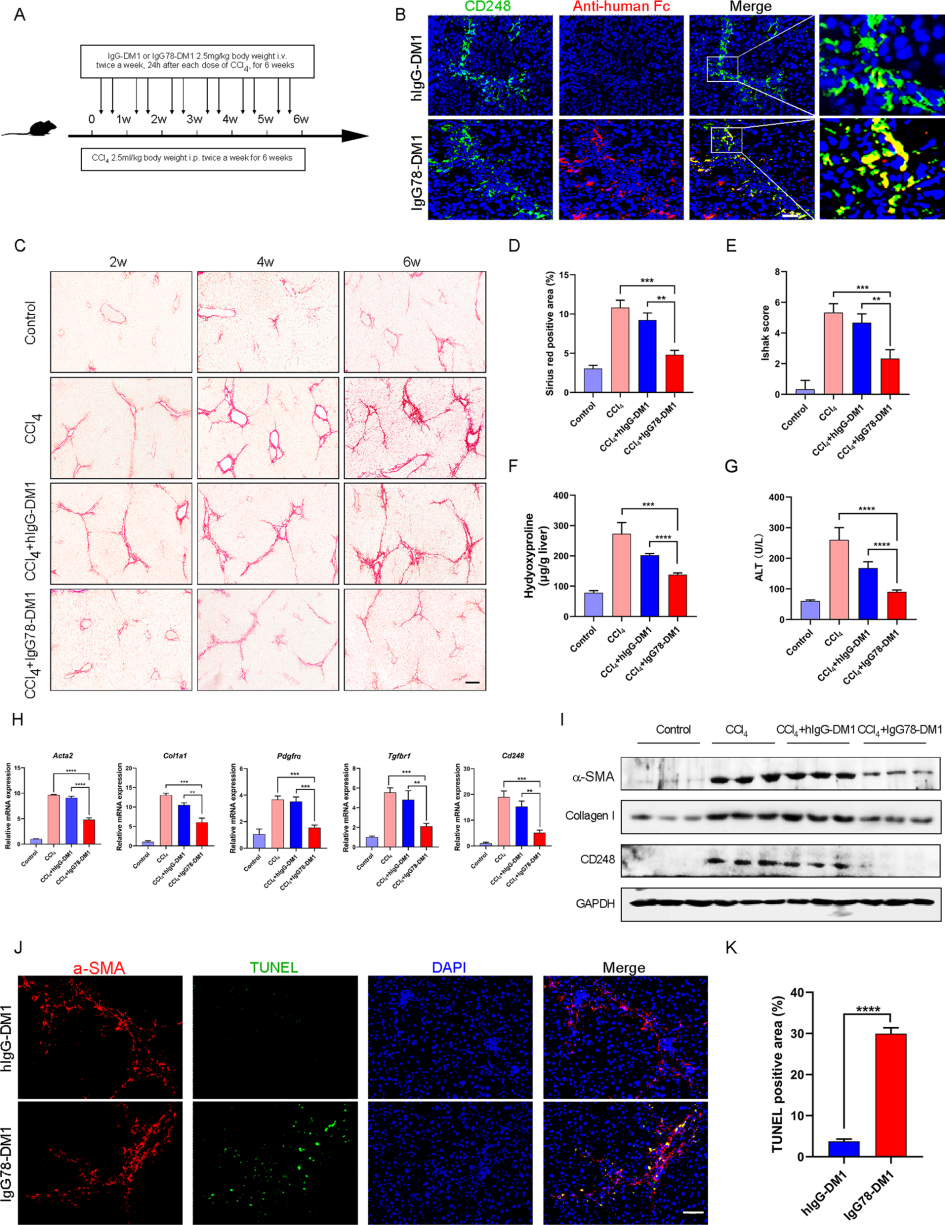
IgG78-DM1 could prevent liver fibrosis in CCl_4_-induced mice. **A** Schematic of the experimental design for the establishment and treatment of CCl_4_-induced mice. **B** IF staining showing the localization of IgG78-DM1 in the liver tissue of CCl_4_-induced mice. **C** Sirius Red staining showing that IgG78-DM1 could alleviate liver fibrosis in CCl_4_-induced mice (n = 5). **D** Quantification of the data in **C**. **E** Ishak score of the liver tissue. **F** The hepatic hydroxyproline content after treatment. **G** Serum levels of ALT after treatment (n = 5 in **B**–**G**). **H** qRT-PCR to show the mRNA levels of *Cd248* and fibrosis-related genes (*Acta2, Col1a1, Tgfbr1 and Pdgfrα*) in the liver tissues. **I** Western blotting showing the protein levels of α-SMA, collagen, and CD248 in liver tissues. **J** TUNEL staining showing the apoptosis of α-SMA-positive HSCs in liver tissues. **K** Quantification of the data in **J** (n = 3 in **H**–**K**). Representative images are shown. Scale bar, 100 μm, **p < 0.01, *** *p* < 0.001, ****p < 0.0001. *DM1* Mertansine, *HSC* hepatic stellate cell, *IF* immunofluorescence, *ALT* alanine aminotransferase, *qRT-PCR* quantitative real-time reverse transcription polymerase chain reaction, *α-SMA* alpha smooth muscle actin, *TUNEL* terminal deoxynulceotidyl transferase nick-end-labeling

Given that IgG78-DM1 could effectively kill CD248^+^ HSCs in vitro, we next examined whether it could alleviate liver fibrosis in vivo. For prophylactic treatment, mice were injected intravenously with IgG78-DM1 or hIgG-DM1 during the process of CCl_4_-induced liver fibrosis (Fig. 5A). We performed pre-experiment to determine the appopriate dosage of IgG78-DM1 for treatment and found 2.5 mg/kg, twice a week could effectively alleviate liver fibrosis. To confirm whether IgG78-DM1 could specifically distribute in liver tissue, we labelled IgG78-DM1 with IRDye 800CW dye and observed its distribution in vivo. Results showed that IgG78-DM1 could specifically enrich in the liver tissue 24 h after injection and fluorescent signal maintained after 96 h (Additional file 4: Fig. S4). To confirm whether IgG78-DM1 could specifically bind with CD248^+^ myofibroblasts in vivo, we treated 6-week CCl_4_-induced mice with IgG78-DM1 for just once and examined the localization of IgG78-DM1 in liver tissue by dual IF staining using frozen sections. The results showed that IgG78-DM1 were mainly colocalized with CD248^+^ cells in vivo (Fig. 5B). Sirius red staining and the Ishak score showed that the deposition of collagen was markedly inhibited after IgG78-DM1 treatment (Fig. 5C–E). Inhibition of liver fibrosis was confirmed by the greatly decreased Masson staining-positive area and reduced α-SMA expression (Additional file 5: Fig. S5A–D). In addition, the content of hydroxyproline in liver tissue and the serum level of alanine aminotransferase (ALT), which indicate the severity of liver fibrosis and impaired liver function, respectively, were decreased markedly in the IgG78-DM1 treatment group, indicating that liver fibrosis was inhibited while liver function was improved (Fig. 5F, G).

The expression levels of CD248 and fibrosis-related factors in liver tissues were then examined using qRT-PCR and western blotting. The results showed that the expression levels of CD248 and fibrosis-related factors [α-SMA, Collagen I, platelet-derived growth factor receptor-alpha (PDGFRα), and transforming growth factor beta receptor 1 (TGF-βR1)] were decreased significantly in the IgG78-DM1 treatment group compared with those in the controls (Fig. 5H and I). To evaluate whether IgG78-DM1 indeed induced specific killing of myofibroblasts in vivo, 6-week CCl_4_-induced mice were treated with IgG78-DM1 or hIgG-DM1 just once and liver tissue was isolated to examine apoptosis using the TUNEL assay. The results showed significantly increased levels of apoptosis of α-SMA^+^ myofibroblasts in the IgG78-DM1 treated group compared with that in the controls (Fig. 5J and K).

**Fig. 6:**
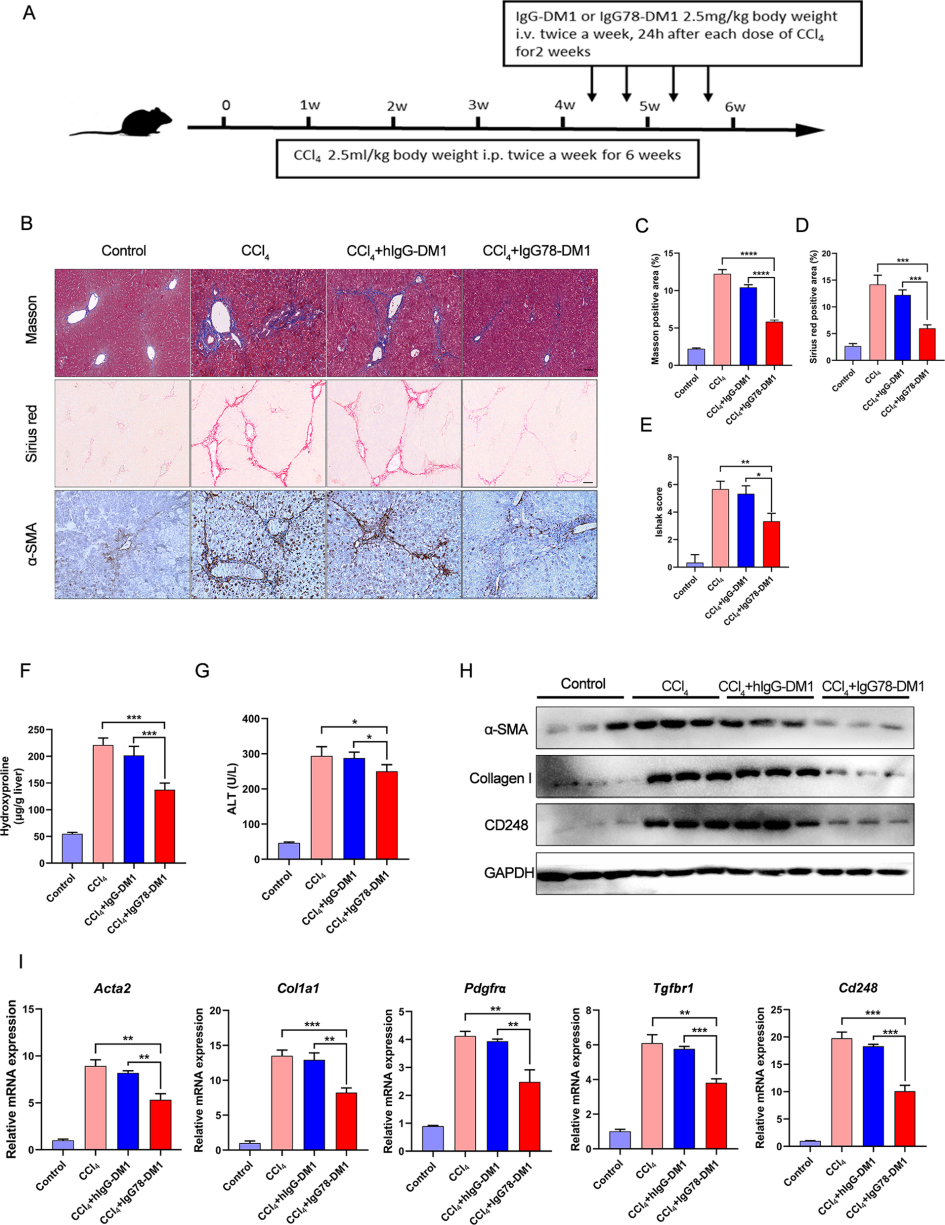
IgG78-DM1 could alleviate liver fibrosis in a therapeutic treatment model. **A** Schematic experimental design for the establishment and treatment of CCl_4_-induced mice. **B** Representative images of Masson staining, Sirius red staining, and IHC staining for α-SMA. **C**, **D** Quantification of the Masson and Sirius red positive area in **B**. **E** Ishak score of the liver tissues. **F** Hepatic hydroxyproline content after treatment. **G** Serum levels of ALT after treatment (n = 5 in **B**–**G**). **H** Western blotting showing the protein levels of α-SMA, collagen I and CD248 in the liver tissues. **I** qRT-PCR to show the mRNA levels of *Cd248* and fibrosis-related genes (*Acta2, Col1a1, Tgfbr1 and Pdgfrα*) in the liver tissues (n = 3 in **H**–**I**). *DM1* Mertansine, *IHC* immunohistochemistry, *ALT* alanine aminotransferase, *α-SMA* alpha smooth muscle actin, *qRT-PCR* quantitative real-time reverse transcription polymerase chain reaction

In addition, to evaluate whether IgG78-DM1 could be used as a therapeutic treatment, we treated the mice with IgG78-DM1 for the last 2 weeks of the 6-week CCl_4_ treatment (Fig. 6A). The results showed that, compared with the hIgG-DM1-treated group, mice treated with IgG78-DM1 showed a significant decrease in Masson and Sirius red positive areas, the Ishak score, and hydroxyproline and serum ALT levels (Fig. 6B–G). Consistently, the expression levels of CD248 and fibrosis-related factors were reduced in IgG78-DM1-treated mice, as assessed using qRT-PCR and western blotting analyses (Fig. 6H, J).

### IgG78-DM1 showed ideal biosafety and reproductive safety in vivo

**Fig. 7:**
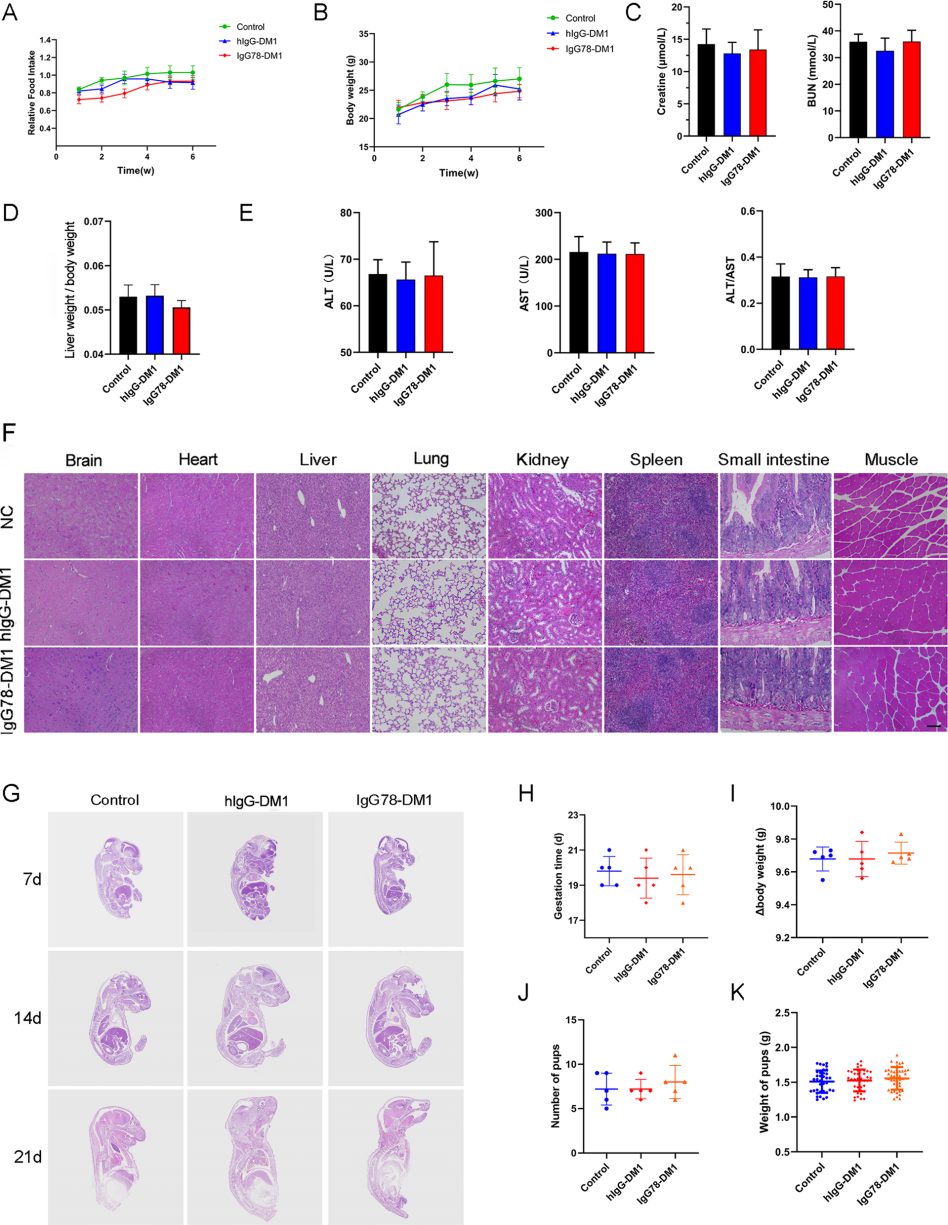
Biosafety and reproductive safety analysis of IgG78-DM1 in C57BL/6 mice. **A**, **B** Food intake and body weight of different groups after IgG78-DM1 or hIgG-DM1 treatment. **C**–**E** Kidney function, liver index (liver weight/body weight), and liver function showing the biosafety of IgG78-DM1 in CCl_4_-induced mice. **F** H&E staining of different organs in CCl_4_-induced mice after IgG78-DM1 or hIgG-DM1 treatment (n = 5 in **A**–**F**). Scale bar, 50 μm. **G** H&E staining of embryos in early, middle, and late stages of pregnancy in normal C57BL/6 mice after IgG78-DM1 or hIgG-DM1 treatment. **H**–**K** Duration of pregnancy, change in body weight during pregnancy, number of offspring, and weight of the pups in each group (n = 5 in **G**–**K**). Representative images are shown. *DM1* Mertansine, *H&E* hematoxylin and eosin

To evaluate the safety of IgG78-DM1 in vivo, a primary safety study was performed in normal C57BL/6 mice. IgG78-DM1 (10 mg/kg) was injected into mice through the tail vein twice a week for 6 weeks; injection of PBS and hIgG-DM1 were used as negative controls. Food intake and the body weight of the mice were analyzed after administration, and no significant difference was observed among the three groups (Fig. 7A and B). At the end of the experiment, all the mice were sacrificed, and the liver of each mouse was weighed to calculate the liver index, for which no obvious difference was found among the three groups. Serum from each mouse was separated to examine indicators of liver and kidney functions, and no obvious toxicity was observed (Fig. 7C–E). In addition, H&E staining revealed that IgG78-DM1 caused no obvious tissue toxicity in important organs, such as the brain, heart, liver, lung, kidney, and spleen, as shown by the relatively normal structure of these organs (Fig. 7F).

We also examined the safety of IgG78-DM1 during embryonic development in normal C57BL/6 mice, because CD248 expression has been reported in several tissues of mouse embryos. Female C57BL/6 mice were mated with male mice and IgG78-DM1 was injected intravenously at 10 mg/kg twice a week from the first day of pregnancy until delivery. No anatomical or histological abnormalities were found in any of the embryos among the different groups during gestation, as shown by the results of H&E staining of the embryos (Fig. 7G). Furthermore, IgG78-DM1 had no obvious influence on gestation time, change of body weight of the pregnant mice, or the number and weight of pups at birth (Fig. 7H–K). These results confirmed that IgG78-DM1 had ideal biosafety and reproductive safety when applied in vivo.

## Discussion

Fibrosis, or excessive tissue scarring, is a common feature of most chronic tissue injuries, among which liver fibrosis is the most common fibrotic disease. However, no antifibrotic therapy has been approved to date. Although several pro-fibrotic cytokines such as TGF-β and PDGF, have been found to play essential roles in the process of fibrosis, systemic inhibition of these cytokines, for example TGF-β, could also impair tumor suppression or cause chronic inflammation because their critical function in normal homeostasis (Dewidar et al. 2019; Friedman et al. 2013). Thus, it is vitally important to explore novel specific anti-fibrotic strategies.

HSCs play an essential role in the progression of liver fibrosis, and activated HSCs are considered an ideal target for anti-fibrotic therapy (Higashi et al. 2017). HSCs are normally considered to be in a quiescent state and do not express α-SMA. After being activated, HSCs will transform into myofibroblasts and the expression of α-SMA and ECM proteins will significantly increase. Several strategies targeting activated HSCs have been designed and evaluated. For example, Zhang et al. (2020) designed pPB peptide-modified, *HMGB1* (high mobility group box 1)-siRNA (small interfering RNA) loaded nanoparticles to alleviate liver fibrosis by inhibiting the activation and proliferation of HSCs. Luo et al. (2019) used chondroitin sulfate nanomicelles (CSmicelles) to target HSCs by binding with CD44, and retinoic acid (RA) and doxorubicin (DOX) were encapsulated to mediate specific cytotoxicity toward HSCs to alleviate liver fibrosis. Bangen et al. (2017) reported that injection of an siRNA targeting the Cyclin E1 mRNA into mice could effectively block Cyclin E1 expression and the proliferation of HSCs, hepatocytes, and leukocytes, resulting in significantly ameliorated liver fibrosis. However, these studies are still in the preclinical stage. More importantly, the lack of a specific target might limit their further application. Therefore, it is important  to identify ideal targets that are specifically expressed on activated, but not quiescent, HSCs (Yazdani et al. 2017).

CD248 is a transmembrane glycoprotein that belongs to C-type lectin-like receptor family (Christian et al. 2001). During development, CD248 is specifically expressed on interstitial fibroblasts and pericytes, but its expression largely disappears in adults (Lax et al. 2007). However, CD248 expression is upregulated on activated HSCs during liver fibrosis, while in *Cd248* knockout mice, CCl_4_-induced liver fibrosis was obviously alleviated and the expression of collagen I, α-SMA, and TGF-β were all inhibited significantly (Mogler et al. 2015; Wilhelm et al. 2016). These findings indicated that CD248 might play an important role in the progression of liver fibrosis and could be an ideal therapeutic target for fibrotic diseases (Teicher 2019).

Recently, it was found that resistance of myofibroblasts to apoptosis played a critical role in fibrotic diseases, and the induction of apoptosis in myofibroblasts could be an effective strategy to alleviate liver fibrosis (Hinz and Lagares 2020; Li et al. 2019, 2020; Oh et al. 2016). In addition, Aghajanian et al. (2019) reported that cardiac fibrosis could be alleviated through specific killing of activated cardiac fibroblasts by FAP-specific CAR T cells, indicating that specific killing of activated fibroblasts could also be an effective way to inhibit tissue fibrosis. Inspired by these findings, we speculated that specifically killing of activated HSCs might have an anti-fibrotic effect in liver fibrosis. To realize specific killing, we generated an ADC named IgG78-DM1, in which the CD248 specific antibody IgG78 was conjugated with the microtubule inhibitor DM1 through an SMCC linker. We examined whether IgG78-DM1 could effectively alleviate CCl_4_-induced liver fibrosis in vivo.

First, we confirmed that CD248 expression was upregulated in the fibrotic liver tissues of both patients with hepatic cirrhosis and in CCl_4_-induced mice. We also confirmed that CD248 was expressed mainly on α-SMA-positive HSCs, which was consistent with previous reports (Mogler et al. 2015; Wilhelm et al. 2016). Next, CD248 expression was demonstrated to be markedly upregulated in activated HSCs, either in TGF-β1 stimulated normal HSCs or in freshly isolated HSCs from 6-week CCl_4_-induced mice. Thus, CD248 was confirmed to be specifically expressed on activated HSCs and could be used as a target to treat liver fibrosis.

To exert specific killing on activated HSCs, ADC IgG78-DM1 was generated. To construct the ADC, the same structure as Tratuzumab-DM1 (T-DM1) was used, in which the antibody was conjugated with DM1 through a non-cleavable SMCC linker (von Minckwitz et al. 2019). CD248-mediated antibody internalization has been reported (Rybinski et al. 2015), and our results demonstrated that IgG78-DM1 could specifically bind with and be internalized into CD248-positive activated HSCs. In addition, IgG78-DM1 could induce specific cytotoxicity of activated HSCs. DM1 is a potent cytotoxic drug; therefore, it could kill target cells effectively, even at very low dose. The use of the non-cleavable SMCC linker to tether DM1 to IgG78 ensured that only CD248-positive activated HSCs were destroyed, while hepatocytes and other normal cells were not affected.

In vivo*,* after IgG78-DM1 was injected into CCl_4_-induced mice, it was distributed specifically in the fibrotic liver and colocalized with CD248 positive cells, as shown by IF staining of frozen liver sections. In addition, IgG78-DM1 alleviated liver fibrosis significantly in both prophylactic and therapeutic treatment models, as shown by the decreased deposition of collagen and decreased expression of fibrosis-related proteins. The results of TUNEL staining indicated that the inhibition of liver fibrosis by IgG78-DM1 was caused by the specific killing of activated HSCs. During our study, we observed that hIgG-DM1 also had some anti-fibrotic effect, which was hypothesized to be caused by metabolism of the ADC in liver tissue. However, the anti-fibrotic effect in IgG78-DM1 treated group was significantly stronger than that of hIgG-DM1.

Besides evaluating the effectiveness of this strategy to alleviate liver fibrosis, we also examined the biosafety and reproductive safety of IgG78-DM1 in vivo. The results of liver and kidney function data and H&E staining of important organs indicated that IgG78-DM1 did not cause obvious tissue toxicity in vivo. In mice, the normal embryo development and the normal gestation time, number, and weight of pups at birth indicated that IgG78-DM1 did not induce reproductive toxicity when applied in vivo.

## Conclusions

In summary, our study demonstrated that CD248 was specifically expressed on activated hepatic stellate cells in liver fibrosis and CD248 could be used as an effective target for anti-fibrotic therapy. A CD248-specific antibody-drug conjugate (IgG78-DM1) was generated that could bind specifically with and kill CD248-positive hepatic stellate cells in vitro, could alleviate liver fibrosis in vivo, and had a good safety profile. To the best of our knowledge, this is the first attempt to alleviate fibrosis using an ADC, and we believe that this strategy could also be applied to treat other fibrotic diseases.

## Supplementary Information

**Additional file 1: Figure S1.** CD248 expression was positively correlated with the severity of fibrosis in patients with liver cirrhosis. (A) Sirius red staining and IHC staining of CD248 in the liver tissue of patients with liver cirrhosis (scale bar, 25 μm). (B) Correlation of CD248 expression and severity of fibrosis in patients with liver cirrhosis. *R*^*2*^ = 0.7161, *p* < 0.0001.

**Additional file 2: Figure S2.** CD248 was expressed in activated HSC cell line JS-1 cells. (A) Flow cytometry showing the expression of vimentin, a marker of myofibroblast, in TGF-β-activated JS-1 cells. (B, C) RT-qPCR and Western blot to show the increased expression of α-SMA, Collagen I and CD248 in TGF-β-activated JS-1 cells. **p < 0.01, ***p < 0.001.

**Additional file 3: Figure S3.** CD248 could not bind with and kill hepatocytes and macrophages. (A, B) Flow cytometry showing the binding of IgG78-DM1 with hepatocytes (A) and macrophages (B). (C, D) CCK8 assay showing the effective killing of hepatocytes (C) and macrophages (D) by IgG78-DM1.

**Additional file 4: Figure S4.** Metabolism of IgG78-DM1 in liver. In vivo imaging showing the enrichment of IgG78-DM1 labelled with IRDye 800CW conjugates 24 h and 96 h after the treatment.

**Additional file 5: Figure S5.** IgG78-DM1 could alleviate liver fibrosis in vivo. (A) Masson staining showing liver fibrosis in CCl4-induced mice after IgG78-DM1 or hIgG-DM1 treatment. (B) Quantitative analysis of the data in A. (C) IHC staining of α-SMA in the liver tissue of CCl_4_-induced mice after IgG78-DM1 or hIgG-DM1 treatment. (D) Quantitative analysis of the data in C. Scale bar, 50 μm. **p < 0.01, ***p < 0.001. n = 5.

## Data Availability

The data and materials of this study are available from the corresponding authors upon request.

## Abbreviations

HSCs: Hepatic stellate cells
ADC: Antibody-drug conjugates
ECM: Extracellular matrix
TGF-β: Transforming growth factor-β
PDGF: Platelet-derived growth factor
α-SMA: α-Smooth muscle actin
AngII/PE: Angiotensin II and phenylephrine
FAP: Fibroblast activation protein
CAR: Chimeric antigen receptor
TEM1: Tumor endothelial marker 1
DM1: Mertansine
