# Supplementary figures and images for: Antibody-drug conjugates targeting CD248 inhibits liver fibrosis through specific killing on myofibroblasts
Source: Mol Med. 2022 Mar 22;28:37. doi: 10.1186/s10020-022-00460-1 (PMC8939076; doi:10.1186/s10020-022-00460-1)

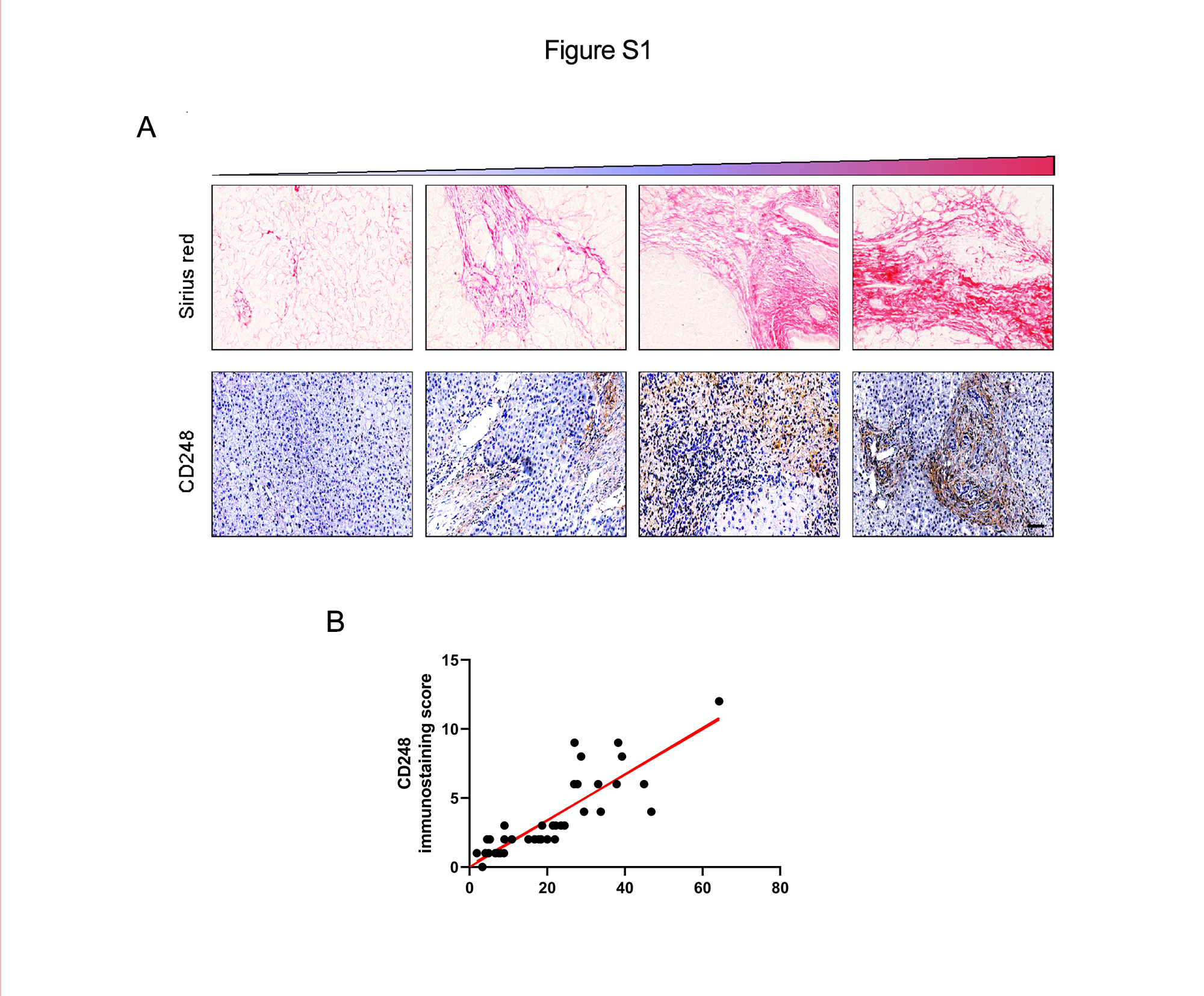

Supplement: Supplementary file 1 — Additional file 1: Figure S1. CD248 expression was positively correlated with the severity of fibrosis in patients with liver cirrhosis. (A) Sirius red staining and IHC staining of CD248 in the liver tissue of patients with liver cirrhosis (scale bar, 25 μm). (B) Correlation of CD248 expression and severity of fibrosis in patients with liver cirrhosis. R2 = 0.7161, p < 0.0001. [file 10020_2022_460_MOESM1_ESM.tif]

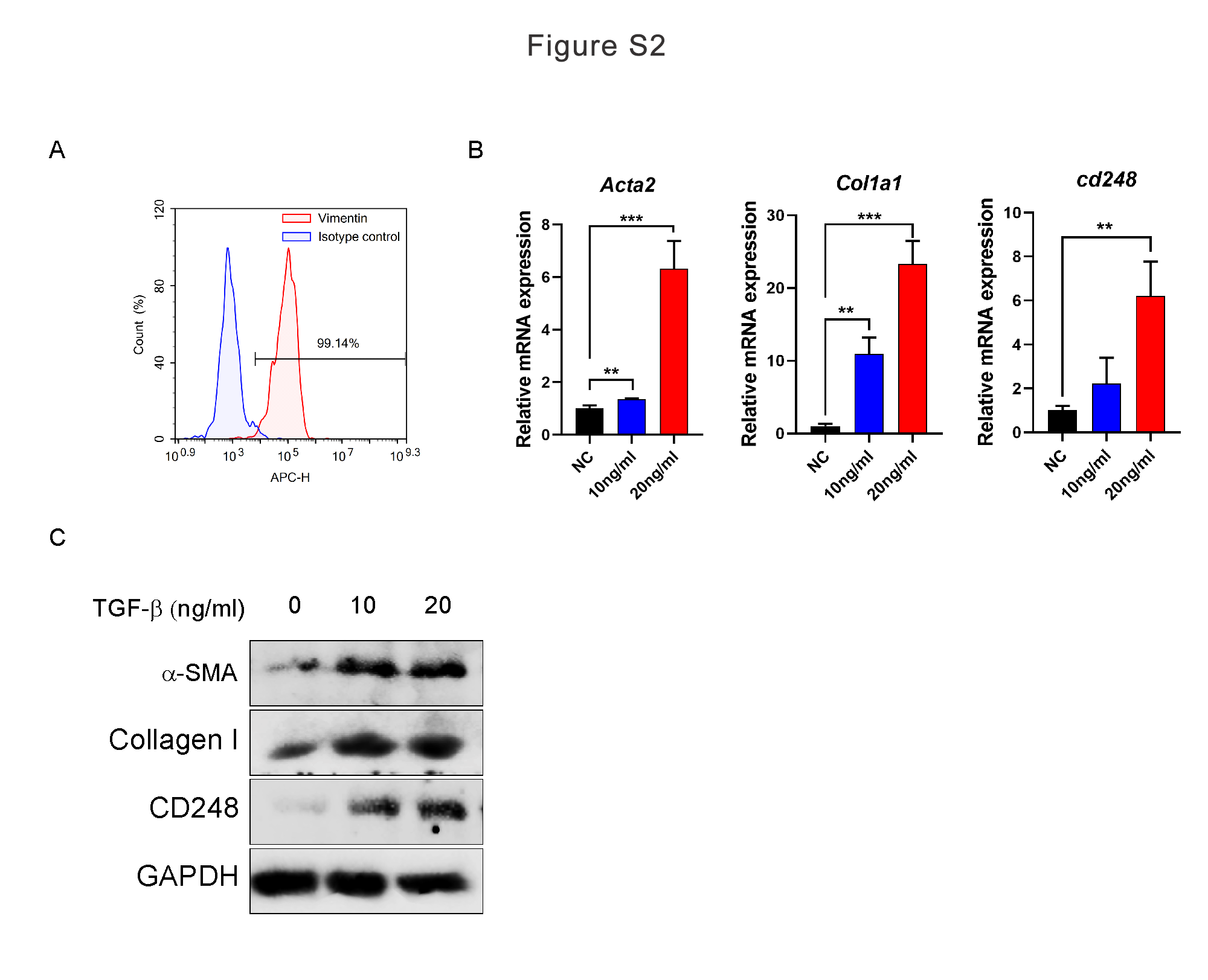

Supplement: Supplementary file 2 — Additional file 2: Figure S2. CD248 was expressed in activated HSC cell line JS-1 cells. (A) Flow cytometry showing the expression of vimentin, a marker of myofibroblast, in TGF-β-activated JS-1 cells. (B, C) RT-qPCR and Western blot to show the increased expression of α-SMA, Collagen I and CD248 in TGF-β-activated JS-1 cells. **p < 0.01, ***p < 0.001. [file 10020_2022_460_MOESM2_ESM.tif]

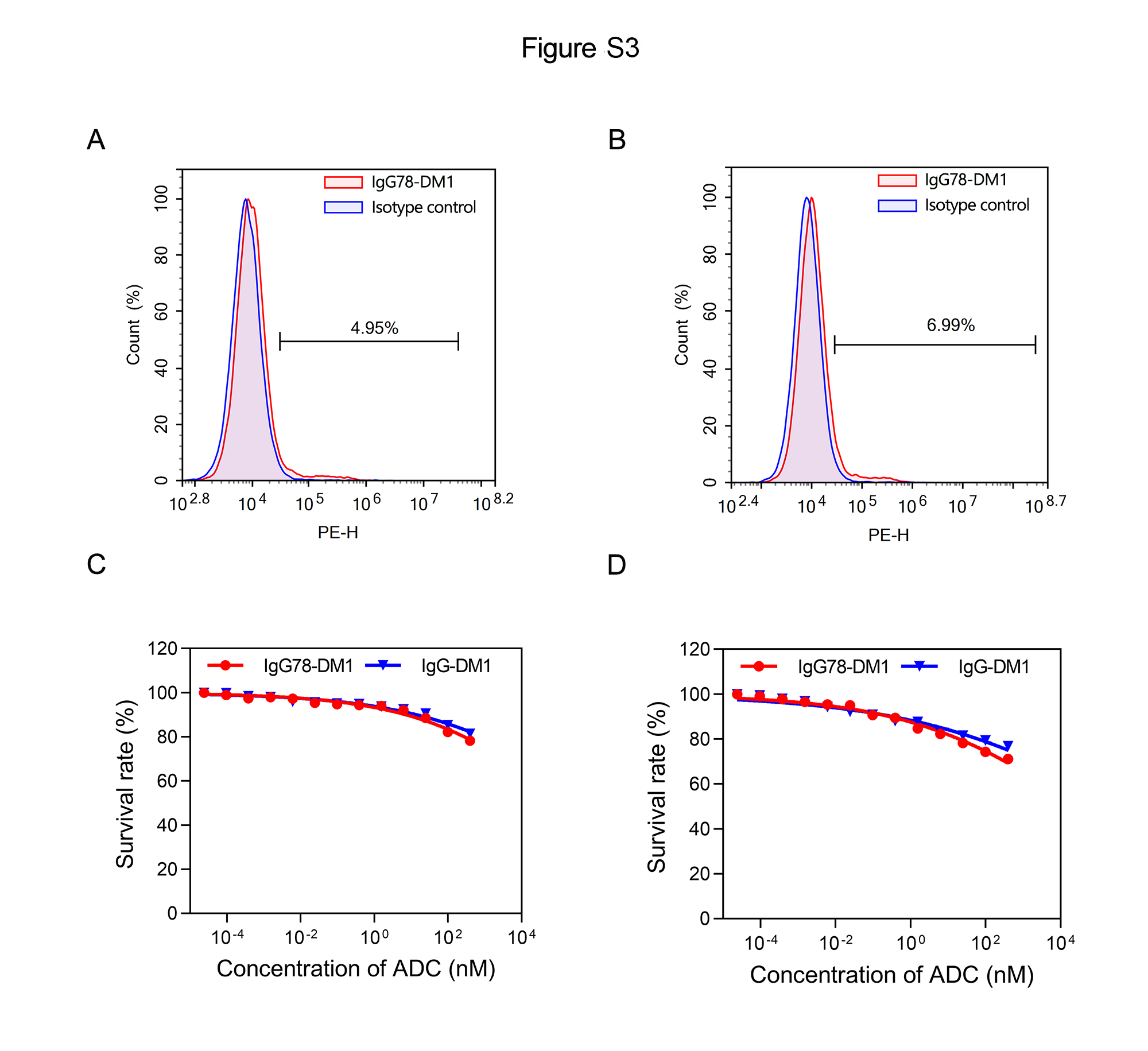

Supplement: Supplementary file 3 — Additional file 3: Figure S3. CD248 could not bind with and kill hepatocytes and macrophages. (A, B) Flow cytometry showing the binding of IgG78-DM1 with hepatocytes (A) and macrophages (B). (C, D) CCK8 assay showing the effective killing of hepatocytes (C) and macrophages (D) by IgG78-DM1. [file 10020_2022_460_MOESM3_ESM.tif]

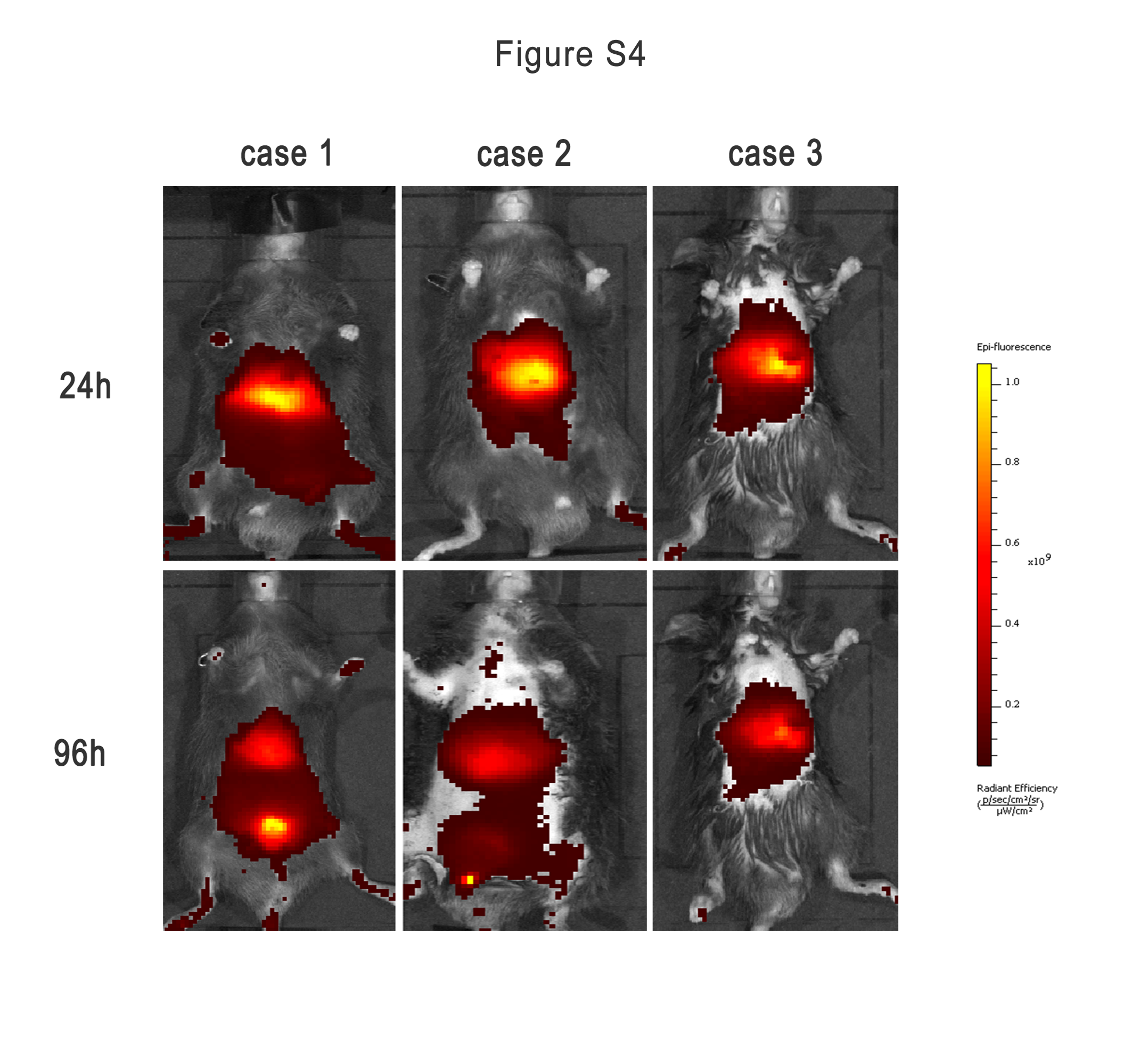

Supplement: Supplementary file 4 — Additional file 4: Figure S4. Metabolism of IgG78-DM1 in liver. In vivo imaging showing the enrichment of IgG78-DM1 labelled with IRDye 800CW conjugates 24 h and 96 h after the treatment. [file 10020_2022_460_MOESM4_ESM.tif]

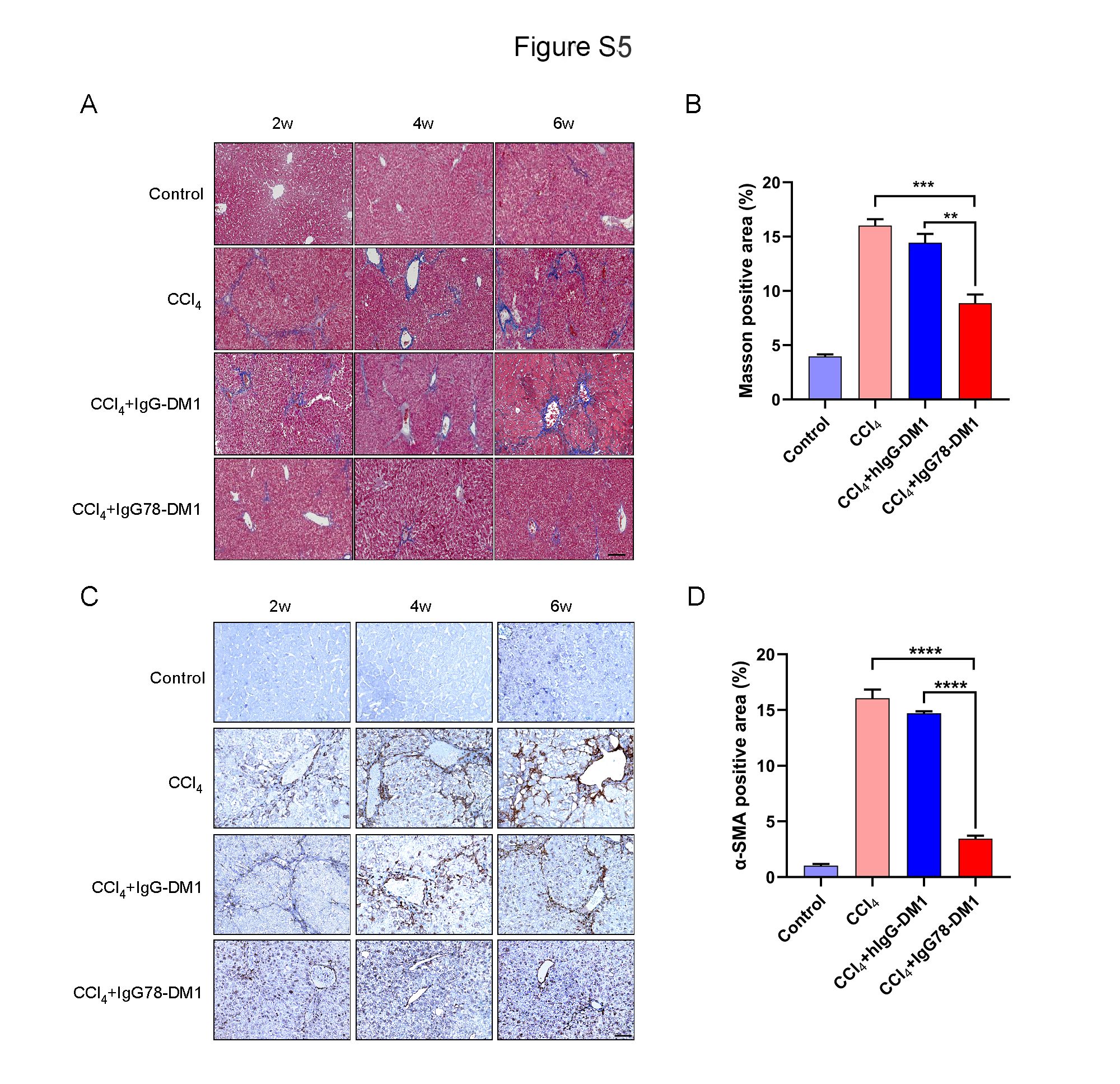

Supplement: Supplementary file 5 — Additional file 5: Figure S5. IgG78-DM1 could alleviate liver fibrosis in vivo. (A) Masson staining showing liver fibrosis in CCl4-induced mice after IgG78-DM1 or hIgG-DM1 treatment. (B) Quantitative analysis of the data in A. (C) IHC staining of α-SMA in the liver tissue of CCl4-induced mice after IgG78-DM1 or hIgG-DM1 treatment. (D) Quantitative analysis of the data in C. Scale bar, 50 μm. **p < 0.01, ***p < 0.001. n = 5. [file 10020_2022_460_MOESM5_ESM.tif]
